# Supplementary material for: Development of chemokine network inhibitors using combinatorial saturation mutagenesis
Source: Commun Biol. 2025 Apr 3;8:549. doi: 10.1038/s42003-025-07778-6 (PMC11969024; doi:10.1038/s42003-025-07778-6)
Supplement: Supplementary file 4 — Supplementary Data 2 [file 42003_2025_7778_MOESM4_ESM.zip › SupplementaryData3/scripts/xattune_ic50.tar!/./script/analysis/JK/JKSuppTableX.docx]

Supplementary Table X - Mean and standard error (SE) of peptide IC50 values

| Chemokine | Peptide | Cells | Replicates | Mean pIC50 [M] | SE pIC50 [M] | Mean IC50 [M] |
| --- | --- | --- | --- | --- | --- | --- |
| ATHERO | HD2CM304 | ATC | 3 | 5.43 | 0.14 | 3.7e-06 |
| ATHERO | HD2 | ATC | 3 | 4.82 | 0.14 | 1.5e-05 |
| ATHERO | HD2CM307 | ATC | 3 | 4.61 | 0.15 | 2.5e-05 |
| ATHERO | HD2CM307 | THP1 | 3 | 4.84 | 0.15 | 1.4e-05 |
| ATHERO | HD2CM304 | THP1 | 3 | 4.44 | 0.04 | 3.6e-05 |
| ATHERO | HD2 | THP1 | 3 | 4.39 | 0.05 | 4.1e-05 |
| INS | HD2CM304 | ATC | 3 | 5.72 | 0.12 | 1.9e-06 |
| INS | HD2CM307 | ATC | 3 | 5.11 | 0.14 | 7.8e-06 |
| INS | HD2 | ATC | 3 | 4.88 | 0.26 | 1.3e-05 |
| INS | HD2CM307 | THP1 | 3 | 5.02 | 0.17 | 9.5e-06 |
| INS | HD2 | THP1 | 3 | 4.54 | 0.14 | 2.9e-05 |
| INS | HD2CM304 | THP1 | 3 | 4.23 | 0.05 | 5.9e-05 |
| RA | HD2CM304 | ATC | 3 | 6.35 | 0.01 | 4.5e-07 |
| RA | HD2 | ATC | 3 | 5.02 | 0.18 | 9.5e-06 |
| RA | HD2CM307 | ATC | 3 | 4.74 | 0.24 | 1.8e-05 |
| RA | HD2CM307 | THP1 | 3 | 4.95 | 0.08 | 1.1e-05 |
| RA | HD2CM304 | THP1 | 3 | 4.77 | 0.13 | 1.7e-05 |
| RA | HD2 | THP1 | 3 | 4.56 | 0.19 | 2.8e-05 |
